# A new approach to selective brain cooling by a Ranque-Hilsch vortex tube

**DOI:** 10.1186/s40635-016-0102-5

**Published:** 2016-09-29

**Authors:** Mohammad Fazel Bakhsheshi, Yong Wang, Lynn Keenliside, Ting-Yim Lee

**Affiliations:** 1 Imaging Program, Lawson Health Research Institute, London, ON Canada; 2 Imaging Research Laboratories, Robarts Research Institute, 1151 Richmond Street North, London, ON N6A 5B7 Canada; 3 Department of Medical Imaging and Biophysics, The University of Western Ontario, London, ON Canada

**Keywords:** Brain temperature, Selective brain cooling, Vortex tube, Intranasal cooling, Physiologic monitoring, Cerebral blood flow

## Abstract

**Background:**

Target temperature management is the single most effective intervention and the gold standard in post-resuscitation care today. However, cooling the whole body below 33–34 °C can cause severe complications. Therefore, developing a selective brain cooling (SBC) approach which can be initiated early to induce rapid cooling and maintain the target temperature over 12–24 h before slowly rewarming brain temperature by itself alone would be advantageous. Vortex tubes are simple mechanical devices generating cold air from a stream of compressed air without applied chemical or energy. This study investigated whether blowing cooled air from a vortex tube into the nasal cavities is safe and effective to selectively reduce and maintain before slowly rewarming brain temperature back to normal temperature.

**Methods:**

Experiments were conducted on ten juvenile pigs. Body temperature was measured using an esophageal and a rectal temperature probe while brain temperature with an intraparenchymal thermocouple probe. Cerebral blood flow (CBF) was measured with CT perfusion.

**Results:**

Brain temperature dropped below 34 °C within 30–40 min while a brain-esophageal temperature difference greater than 3 °C was maintained over 6 h. There was no evidence of nasal or nasopharynx mucosal swelling, necrosis, or hemorrhage on MRI examination. CBF first decreased and then stabilized together with brain temperature before increasing to the baseline level during rewarming.

**Conclusions:**

SBC was accomplished by blowing cold air from a vortex tube into the nasal cavities. Due to its portability, the method can be used continuously in resuscitated patients in both in- and out-of-hospital situations without interruption.

## Background

The global incidence of sudden cardiac arrest is around 3.7 million deaths every year [1]. In the USA alone in 2013, approximately 359,400 people experienced out-of-hospital cardiac arrests, in which less than 9.5 % survived. In contrast, approximately 209,000 cardiac arrests occur each year in hospitals, and 23 % of those patients survive [2]. Of those patients who survive after out-of-hospital cardiac arrest, about two thirds died due to neurological injury [3]. Despite advances in the treatment of cardiac arrest, there is a great need for technology that improves patient outcomes. Target temperature management has been shown to improve survival and neurologic recovery after cardiac arrest [4, 5]. Analysis of survival at different time points after cardiac arrest shows that ~40 % of resuscitated patients return successfully to spontaneous circulation and could therefore be eligible for target temperature management [6, 7]. However, current cooling methods are far from optimal. Most brain cooling methods rely on cooling down the whole body primarily using surface cooling devices and invasive intravascular cooling devices; however, decreasing the whole body temperature below 34 °C can induce severe complications. Dysrhythmias, infections, and primary coagulopathy are the most commonly noted complications [8]. Therefore, selective brain cooling methods have been investigated to minimize the complications associated with systemic hypothermia by selectively cooling the brain while maintaining normal core body temperature [9]. However, these solutions suffer from insufficient cooling, insufficient localization of cooling, use of a relatively expensive coolant, and/or irritant effects on skin contact points [10]. Accordingly, we believe that there is a need for an effective, non-invasive, and portable device, with the capability to rapidly induce brain hypothermia in a hospital or field emergency setting and maintain it over an extended period of time before slowly rewarming brain temperature back to normal temperature [4, 5, 11–14].

Vortex tubes, also known as Ranque-Hilsch vortex tubes, are very simple mechanical devices to separate a stream of compressed air into a cold and a hot stream without any moving parts. The vortex tube was first discovered by a French physicist named Georges J. Ranque in 1931 [15] and later improved by a German engineer Rudolf Hilsch in 1947 [16]. Vortex tubes have been used in different applications as they are compact, simple to operate, and require no electrical power or chemical agent. They are typically used in cooling machine parts, cutting tools, spots under thermal stresses, or enclosed electric or electronic control cabinets; they are also used in refrigeration, solidifying polymers, liquefying natural gas, separating mixed gases, and controlling ambient air temperature [17, 18].

In a recent paper [19], we showed the potential use of the vortex tube to selective lowering of brain temperature using various sources of compressed air. In this study, we investigated whether blowing cooled air (−5 to 16 °C) produced by a vortex tube into the nasal cavities is an effective cooling method to selectively reduce and maintain brain temperature over an extended period of time before gradually returning to the baseline temperature on normal juvenile pigs. Cooling the nasal cavities may offer the capacity to cool the brain selectively due to anatomic proximity of the internal carotid artery to the cavernous sinus. Furthermore, cerebrospinal fluid (CSF) chilled at the basal cistern cools the whole brain through the CSF circulation. Although it is unlikely that air at subzero temperature will induce freezing damage to the mucosa, embedded blood vessels, and nerves of the nasal cavities, we used magnetic resonance imaging (MRI) to examine the nasal cavities for tissue damage in three pigs following ~7 h of cooling. Furthermore, we investigated the effect of the proposed selective brain cooling (SBC) method on cerebral blood flow (CBF) measured by CT perfusion [20].

## Methods

### Animal preparation and experimental procedure

Experiments were conducted on ten juvenile Duroc × Landrace crossbred pigs, approximately 2–3 months old with a weight of 29 ± 2 kg. The number of animals used in our experiments was determined based on the similar pilot studies and review of the literature carried out by us [21–23]. All animal experiments were approved by the Animal Use Subcommittee of the Canadian Council on Animal Care at our institution. The methods were carried out in accordance with the approved guidelines. Pigs were anesthetized with 3–4 % isoflurane during preparatory surgery. The animal was intubated with a cuffed endotracheal tube and ventilated with a volume-controlled mechanical ventilator to deliver oxygen/medical air mixture (2:1). A femoral artery was catheterized to monitor heart rate (HR) and mean arterial blood pressure (MAP) and to intermittently collect arterial blood samples for gas (*p*_*a*_CO_2_, *p*_*a*_O_2_, *S*_*a*_O_2_), electrolyte (*cNa*^+^, *cK*^+^, *cCl*^−^, and *cCa*^2+^), pH, and glucose analysis. A cannula was inserted into an ear vein for infusion of propofol (AstraZeneca Pharmaceuticals Canada Inc.). Arterial CO_2_ tension (*p*_*a*_CO_2_) was maintained at normocapnia between 37 and 42 mmHg by adjusting the breathing rate and volume. End-tidal CO_2_ tension (EtCO_2_), tidal volume, respiratory rate, pulse oximetry (SpO_2_), and HR were continuously measured using a multiparameter monitor (SurgiVet Advisor Vital Signs Monitor V9200, Smiths Medical, Dublin, OH, USA). Blood glucose was monitored intermittently, and if it fell below 4.5 mmol/L, a 1–2 mL infusion of 25 % dextrose solution was administered intravenously. Arterial blood gases were measured every hour during the cooling and maintenance phases, by blood gas analyzer (ABL80 FLEX CO-OX, Radiometer Medical ApS, DK-2700, Brønshøj, Denmark), and maintained within the normal range during the experimental period.

Body temperature was measured continuously using an esophageal and a rectal temperature probe attached to the same SurgiVet monitor (temperature probe WWV3418, Smiths Medical, Dublin, OH, USA). Brain temperature was also measured continuously with a thermocouple probe. A 15-mm burr hole was drilled in the skull 1.5 cm posterior and 1.5 cm lateral to the bregma along the midline with a Dremel tool. The needle thermocouple probe was inserted through the burr hole into the brain to a depth of ≈2 cm from the brain surface to measure brain temperature.

**Fig. 1:**
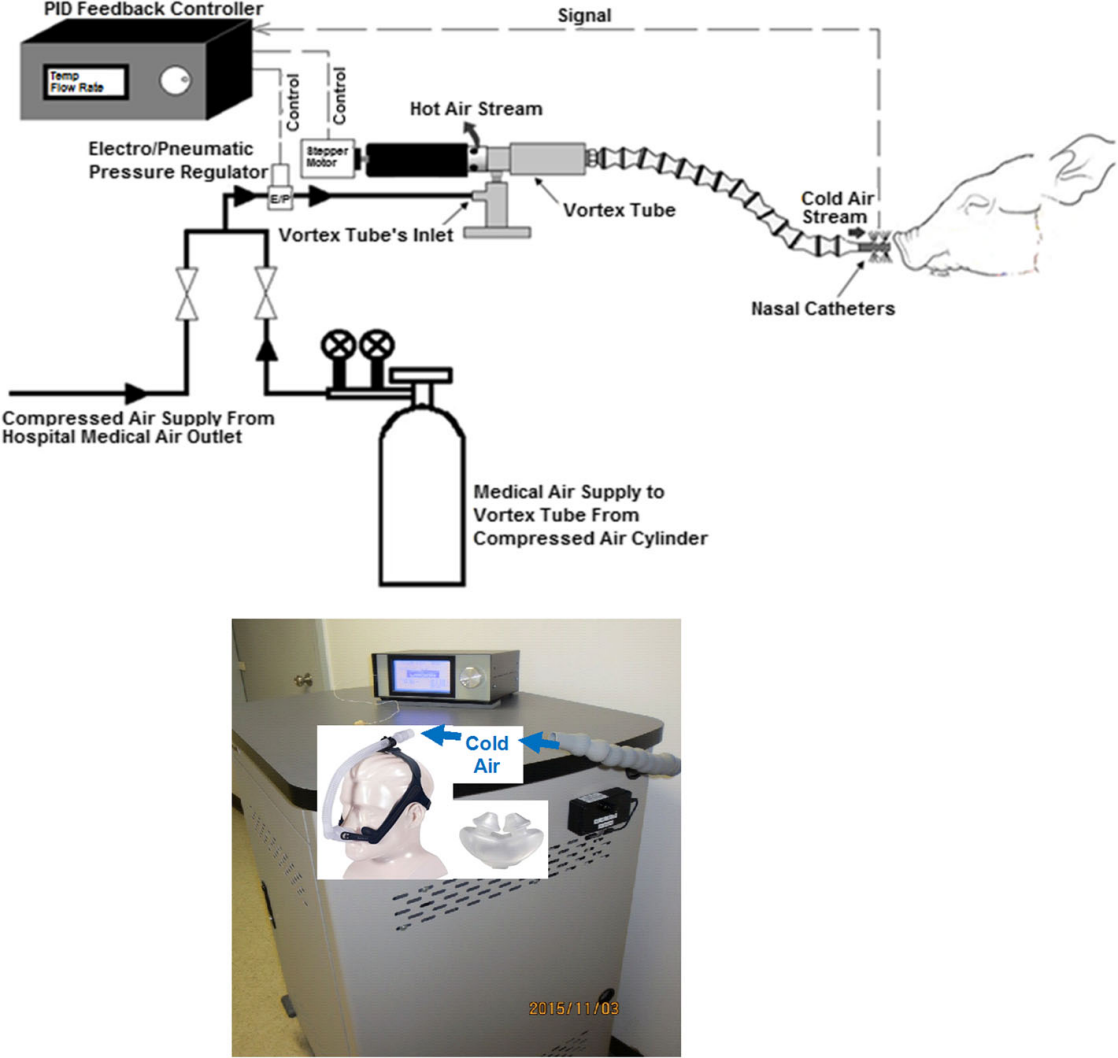
Schematic drawing of the cooling circuit used for intranasal brain cooling. Also, image shows a clinical prototype

Following surgery, each pig was placed prone on the couch of a 64-slice CT scanner (GE Healthcare, Waukesha, WI, USA) and wrapped together with a heated recirculating water pad in a linen blanket. Anesthesia was maintained by ventilation with isoflurane (0.5–1.5 %) and intravenous infusion of propofol (30–50 mL/h and 10 mg/mL). We supplemented isoflurane for anesthesia with propofol to avoid excessive activation of K_ATP_ channels leading to hyperkalemia [24]. A period of at least 30–45 min was allowed for stabilization of physiological conditions before intranasal cooling was initiated with the vortex tube-based cooling apparatus (Fig. 1). The isoflurane concentration and propofol infusion rate were adjusted according to the change in vital signs such as blood pressure, HR, electrolyte levels, and pain responsiveness of the pig.

After the stabilization period, intranasal brain cooling was initiated by blowing cold air (−3 ± 2 °C) at a flow rate of 40–50 L/min into both nostrils for 50–60 min. Once the brain temperature stabilized at the target temperature of 34 ± 1 °C as measured by the intracranial thermocouple, the flow rate was decreased to 30–40 L/min and air temperature increased to 1 ± 3 °C to maintain the target temperature for 6 h while core body temperature (i.e., rectal and esophageal temperatures) was maintained above 36 °C using the heated recirculating water pad and by packing gloves filled with hot water around the body of the pig within the linen blanket. After 6 h of cooling, the brain temperature was allowed to gradually return to the baseline temperature in 2.5 h by increasing the air temperature to 14 ± 2 °C and adjusting the flow rate to 10–30 L/min. Active warming was performed during both maintenance and rewarming periods. Each experiment was completed within 13–14 h, and the animal was sacrificed with intravenous potassium chloride (1–2 mL/kg, 2 mEq/mL) infusion.

### Method of intranasal brain cooling

The commercially available vortex tube (adjustable cold air gun, ITW Vortec Ltd.) is a compact and simple mechanical device that can produce cold air at different temperatures and flow rates from a stream of compressed gas without any moving parts, chemical reactions, or external energy supply. The source of compressed air was either medical air cylinders supplied by L’Air Liquide Ltd. (St. Thomas, ON, CA), with a capacity of 6569 L at fill pressure of 15,617 kPa downregulated to 344 kPa, or a hospital medical air outlet at fixed outlet pressure of 344 kPa. High-pressure compressed air from either source was applied to the inlet nozzle of an electropneumatic pressure regulator (PULSTRONIC II series 605, Numatics Inc.) to monitor and precisely regulate the pressure of compressed air before entering the vortex tube. The compressed air then passed through a generation chamber (inside the vortex tube) which created the vortices inside the tube and caused the compressed air flow to be separated into a cold and a hot stream traveling in opposite directions in the tube. The fraction of compressed air exiting as cold air (also referred as cold fraction ratio) was adjusted by a throttle needle valve via a stepper motor (IMDE17-M Integrated Motor/Driver, RMS Technologies Inc., NV, USA). Both temperature and flow rate of the cold air stream were controlled and monitored continuously by a microprocessor-based digital controller that included a feedback system (Atmel’s AVR Microcontroller, ATmega64A 8-bit with 64K bytes in-system programmable flash). It automatically regulated the inlet pressure and fraction ratio based on the desired cold output air temperature and flow rate from the vortex tube to tightly control the brain temperature and rewarming rate throughout the experiments. Moreover, the temperature of air exiting the cold air outlet of the vortex tube was monitored and recorded continuously with a thermometer (Thermometer/Data Logger, HH309A, with Four Type K Thermocouple Inputs, Omega Engineering, Stamford, CT; resolution 0.1 °C). Intranasal brain cooling was achieved by connecting two nasal catheters (made from polyvinyl chloride, PVC) to the tube from the cold air outlet of a vortex tube. The catheters were coated with 2 % lidocaine gel for local anesthesia and ensuring their better contact with turbinates in the nasal cavity and were inserted 8–10 cm into each nostril. A thermistor was also placed inside one of the two nasal catheters to monitor temperature inside the nasal cavity throughout the experiments. Figure 1 shows the schematic of the experimental setup.

### CT perfusion study for measurement of CBF

In seven pigs, following the baseline (normothermia) CT perfusion study, repeat studies were collected every 2 h throughout the cooling and rewarming phases. All CT perfusion studies were acquired with a GE Healthcare VCT 64-slice CT scanner. Each CT perfusion study began with a scout CT scan to choose the locations of the CT slices to be included in the study. For the study, each pig received a 1.0 mL/kg injection of the iodinated contrast agent iohexol (370 mg I/mL; Isovue™, GE Healthcare, Waukesha, WI) at a rate of 3.0 mL/s into the cephalic vein. Sequential (dynamic) CT scans were acquired using 80 kVp and 200 mA once every second for a period of 40 s. Each study provided eight contiguous 5-mm-thick coronal slices with a 16-cm field of view set to encompass the entire head of the pig. CBF map of each slice was generated from the set of dynamic images using CT Perfusion software (GE Healthcare). Using an in-house software package developed in the IDL Development Environment (ITT Visual Information Solutions, Boulder, CO), regions of interest (ROIs) were manually drawn to encompass the whole brain on each of the CBF maps to read out the average CBF within the ROIs.

### MRI data acquisition

Following a cooling and maintenance period, to assess the impact of nasal cooling on the upper respiratory airway, three pigs underwent an MRI scan using a 3-T MR scanner (Biograph PET/MR scanner, Siemens Medical Systems, Erlangen, Germany) equipped with eight-channel head array coils. The MRI sequences included a 3D T1-weighted gradient-recalled echo sequence (repetition time (TR)/echo time (TE), 2000 ms/3.1 ms; slice thickness, 1 mm; field of view (FOV), 22 × 22 cm; matrix, 256 × 256), a T2-weighted spin echo sequence (TR/TE, 6100 ms/99 ms; slice thickness, 5 mm; FOV, 15 × 15 cm; matrix, 448 × 314), a fat-suppressed T2-weighted sequence (turbo inversion recovery magnitude, TR/TE, 4780 ms/41 ms; FOV, 15 × 15 cm; slice thickness, 5 mm; matrix, 320 × 224), and a fluid-attenuated inversion recovery sequence (TR/TE, 9000 ms/96 ms; FOV, 13.6 × 12.3 cm; slice thickness, 5 mm; matrix, 256 × 232). Diffusion-weighted imaging was acquired using echo planar technique with the following parameters: TR/TE, 12,500 ms/85 ms; FOV, 20.6 × 20.6 cm; section thickness, 5 mm; matrix, 160 × 160, *b* values 0 and 1000 s/mm^2^), and apparent diffusion coefficient (ADC) maps were calculated automatically by the MRI software. The acquisition duration for each combination was 15 min. The MRI images before and after 7 h of intranasal cooling were evaluated by a radiologist (10 years’ experience).

### Statistical analysis

SPSS 17.0.0 (SPSS, Inc., Chicago, IL) was used for all statistical analyses. Monitored physiologic parameters and vital signs were analyzed by repeated measures ANOVA followed by post hoc test with Bonferroni correction to determine statistical differences at different times within a group and between groups at different times. The within-subject variance is assumed constant, and observations within the subject are independent. Statistical significance was based on *p* value <0.05. All data are presented as mean ± standard deviation (SD) unless otherwise noted.

## Results

### Physiological parameters measured at different brain temperatures during selective brain cooling

Table 1 displays a summary of the mean ± SD over ten pigs of each measured physiological parameters prior to cooling, at different times in the cooling, maintenance (at the target temperature of 33 ± 1 °C), and rewarming phases. HR and mean arterial blood pressure (MAP) dropped slowly for the first 2 h after the induction of intranasal cooling from 126 ± 31 to 91 ± 7 (*p* < 0.041) and from 74 ± 8 to 55 ± 6 mmHg (*p* < 0.002), respectively, then stabilized for the rest of the cooling period. No instance of arrhythmia was noted during the cooling or rewarming phases. Other measured physiologic parameters were stable and within normal limits throughout the experiment.

**Table 1:** Physiological parameters (mean ± standard deviation (SD)) measured at different brain temperatures at baseline and during cooling and rewarming; SD was not shown if it was less than the last digit shown in the mean

	Baseline	Cooling and maintenance phases							Rewarming phase		
	1–45 min	1 h	2 h	3 h	4 h	5 h	6 h	7 h	1 h	2 h	3 h
Brain temp (°C)	38.1 ± 0.7	34.2 ± 1.1*	33.5 ± 0.4*	33.4 ± 0.6*	33.3 ± 0.6*	33.5 ± 0.7*	33.4 ± 0.4*	33.2 ± 0.6*	34.8 ± 0.3*	36.2 ± 0.2*	37.6 ± 0.3
S_a_O_2_ (%)	100	100	100	100	100	100	100	100	100	100	100
P_a_O_2_ (mmHg)	200 ± 51	225 ± 51	211 ± 53	213 ± 60	216 ± 67	210 ± 61	206 ± 51	200 ± 66	205 ± 52	204 ± 48	223 ± 36
cNa^+^ (mmol/L)	141 ± 2	140 ± 2	140 ± 2	140 ± 2	140 ± 2	139 ± 2	140 ± 2	139 ± 2	139 ± 2	139 ± 2	138 ± 2
cK^+^ (mmol/L)	4.5 ± 0.4	4.5 ± 0.3	4.8 ± 0.5	5.0 ± 0.6	4.9 ± 0.6	4.9 ± 0.6	4.9 ± 0.6	4.8 ± 0.6	4.8 ± 0.6	4.9 ± 0.7	4.9 ± 0.6
cCa^2+^ (mmol/L)	1.3	1.4	1.4	1.4	1.3	1.3	1.3	1.3	1.3	1.3	1.3
cCl^−^ (mmol/L)	103 ± 2	103 ± 3	103 ± 3	103 ± 3	103 ± 2	102 ± 2	102 ± 3	102 ± 2	101 ± 2	100 ± 2	101 ± 2
MAP (mmHg)	74 ± 8	60 ± 5*	55 ± 6*	56 ± 6*	58 ± 9*	58 ± 10*	61 ± 8*	63 ± 11*	65 ± 11	66 ± 2	65 ± 3
HR (bpm)	126 ± 31	96 ± 9	91 ± 7*	89 ± 3*	90 ± 7*	88 ± 5*	88 ± 3*	92 ± 3*	92 ± 3*	91 ± 6*	93 ± 5*
pH	7.5	7.5	7.5	7.5	7.5	7.5	7.5	7.5	7.5	7.5	7.5
tHb (g/dL)	9.1 ± 0.6	9.2 ± 0.4	9.2 ± 0.7	9.1 ± 0.5	9.1 ± 0.7	8.8 ± 0.9	9.2 ± 0.8	9.1 ± 0.8	8.7 ± 0.9	8.9 ± 0.6	9.0 ± 0.6
P_a_CO_2_ (mmHg)	39 ± 2	37 ± 3	37 ± 2	40 ± 1	38 ± 2	40 ± 1	38 ± 2	38 ± 2	40 ± 2	40 ± 2	41 ± 1
CBF (mL/100 g/min)	37 ± 2	N/A	25 ± 2	N/A	25 ± 3	N/A	28 ± 3	N/A	N/A	N/A	39 ± 4

Omnibus ANOVA and post hoc Tukey test were used to detect changes of each monitored parameter over time

*A statistically significant (*p* < 0.05) change compared to baseline

*SaO*_*2*_ oxygen saturation, *P*_*a*_ *O*_*2*_ partial pressure of oxygen, *cNa* ^*+*^ sodium concentration, *cK* ^*+*^ potassium concentration, *cCa* ^*+*^ calcium concentration, *cCl* ^*−*^ chloride concentration, *tHb* total hemoglobin concentration, *P*_*a*_ *CO*_*2*_ partial pressure of carbon dioxide were measured in arterial blood samples, *MAP* mean arterial pressure, *HR* heart rate were measured from a femoral artery catheter, *CBF* cerebral blood flow, *N/A* not available data

### Brain, rectal, and esophageal temperatures versus time for intranasal cooling method

**Fig. 2:**
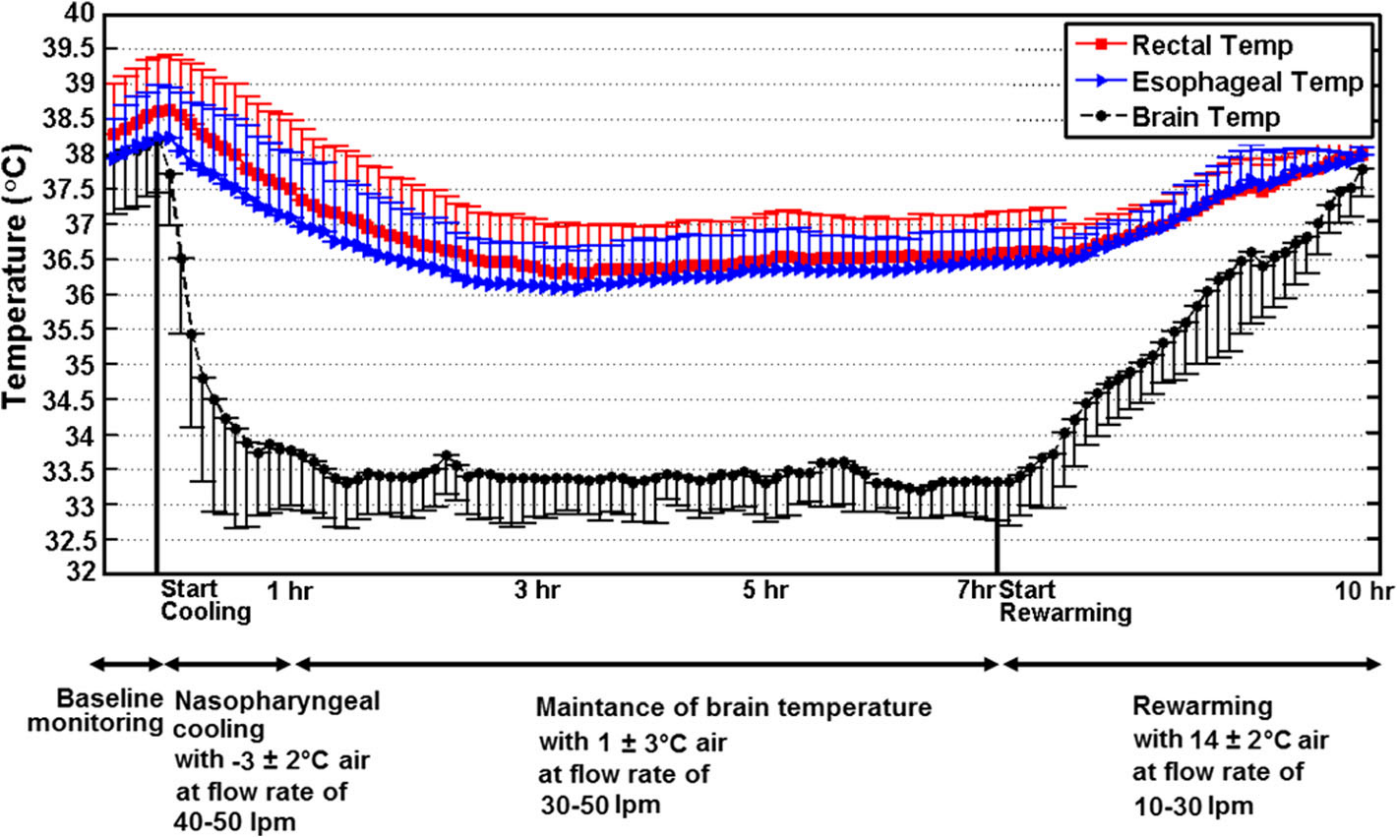
Measured brain, rectal, and esophageal temperatures over time during the baseline, cooling, and rewarming phases. Data were obtained from ten pigs during baseline and in the cooling phase and from seven pigs in the rewarming phase

Mean brain, rectal, and esophageal temperatures versus time are shown in Fig. 2. During 40–60 min of baseline monitoring, mean brain and core body temperatures did not increase more than 0.2 ± 0.1 °C. The pigs had an average brain and core body temperature of 38.2 ± 0.7 °C and 38.6 ± 0.8 °C, which was within the normal temperature range, respectively [25]. Following baseline, intranasal cooling was initiated with −3 ± 1 °C air at a flow rate of 40–50 L/min. Brain temperature decreased biexponentially, dropping rapidly to 34.0 ± 1.4 °C within 30 min and then decreased more slowly and stabilized at 33.7 ± 0.8 °C within the first hour of brain cooling resulting in a mean brain cooling rate of 4.5 ± 0.8 °C/h. The rectal and esophageal temperature decreased during the same interval from 38.6 ± 1.1 °C and 38.2 ± 0.8 °C to 37.3 ± 0.9 °C and 36.9 ± 0.9 °C corresponding to cooling rates of 1.3 ± 0.2 °C/h and 1.3 ± 0.1 °C/h, respectively. After the first hour, the brain temperature could be maintained at 33.4 ± 0.3 °C for another 6 h by increasing the air temperature to 1 ± 3 °C and lowering the flow rate to 30–50 L/min, while both esophageal and rectal temperatures remained above 36.6 ± 0.3 °C which was within the normothermia range. As shown in Fig. 2, after the brain temperature stabilized at 33.4 ± 0.3 °C, the esophageal and rectal temperatures continued to decrease for another hour before stabilizing at 36.6 ± 0.3 °C. The brain-body temperature gradient calculated as the difference between brain and esophageal temperatures peaked at 3.9 ± 0.9 °C but stabilized to 3.2 ± 0.1 °C at 40 and 60 min into cooling, respectively.

After 7 h of cooling, rewarming was initiated by wrapping recirculating hot water blanket and packing hot water pads around the body of the pig and blowing 14 ± 2 °C air at a flow rate of 10–30 L/min into the nostrils. During this phase, the brain and esophageal temperatures increased by 1.7 ± 0.2 °C/h and 1.3 ± 0.2 °C/h, respectively. No difference was found in the rewarming rate as measured by either the rectal or esophageal temperature probe. No abnormalities were noted during the rewarming period.

### CBF measurements using CT perfusion technique

**Fig. 3:**
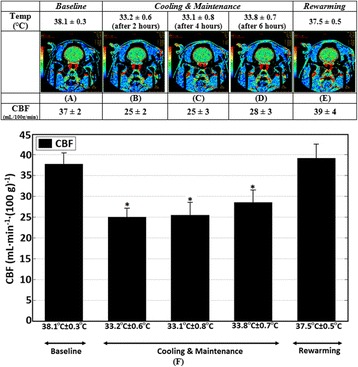
a–e Coronal cerebral blood flow maps of the same coronal, 5-mm-thick slice of a pig brain at baseline, during cooling, and at rewarming. Also shown are regions of interest outlined in *red* used to calculate the average cerebral blood flow (CBF) within the slice. f CBF at each brain temperature. Values are shown as mean ± SD; *p < 0.05 versus baseline CBF

Figure 3a–e shows CBF maps in a pig for the same coronal, 5-mm-thick brain slice and the ROI used to calculate the average CBF within the slice at different brain temperatures. As shown in Fig. 3f, as brain temperature decreased from baseline (38.1 ± 0.2 °C) to 33.3 ± 0.6 °C, CBF also decreased from 37 ± 2 to 26 ± 3 mL/min (100 g)^−1^ (*p* < 0.0001). CBF remained relatively stable throughout the rest of the cooling period; however, during rewarming, when the brain temperature reached 37.5 ± 0.5 °C, CBF increased from 26 ± 3 to the baseline value of 39 ± 4 mL/min (100 g)^−1^.

### Examination of the nasal cavities to assess for tissue damages using MRI

**Fig. 4:**
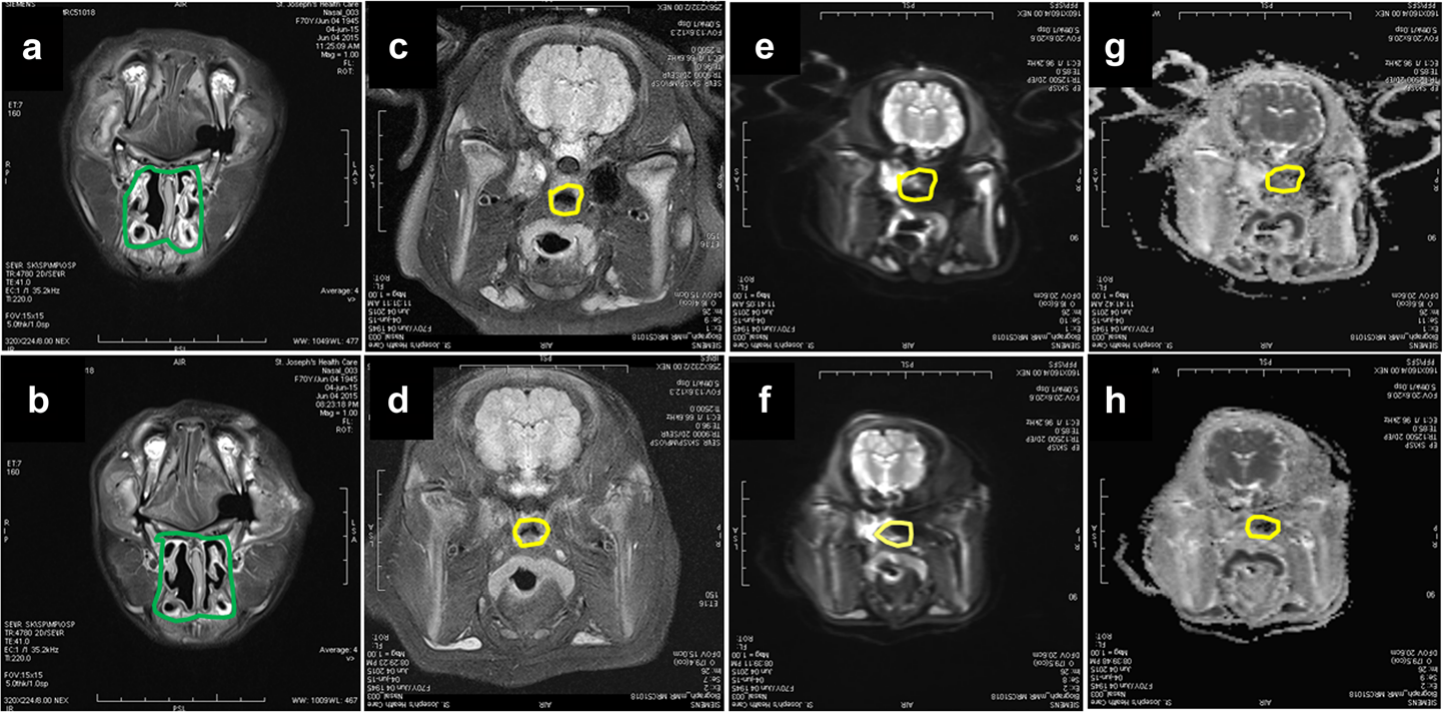
Coronal head MRI of a pig before and after intranasal cooling. **a**, **b** Coronal fat-suppressed T2-weighted MRI of the nasal cavities (*green* outlined region of interest (ROI)) before and after 7 h of cooling. **c**, **d** Coronal fluid-attenuated inversion recovery (FLAIR) MRI of the nasopharynx (*yellow* outlined ROI) and the brain before and after 7 h of cooling. **e**, **f** Diffusion-weighted imaging (DWI) of the nasopharynx (*yellow* outlined ROI) and the brain before and after 6 h of cooling. **g**, **h** Apparent diffusion coefficient (ADC) maps of the nasopharynx (*yellow* outlined ROI) before and after 7 h of intranasal cooling

The MRI images before and after 7 h of intranasal cooling are shown in Fig. 4. There were no engorged turbinates, mucosal swelling, or excessive secretions visible on MRI imaging; and there were no significant changes in the DWI or ADC image intensity values of the nasopharynx area before and after intranasal cooling. The fluid-attenuated inversion recovery (FLAIR) brain images before and after cooling also were normal.

## Discussion

Selective brain cooling in the brain can be achieved via two distinct mechanisms: (1) direct surface cooling of superficial venous blood which in turn cools the blood in the brain via diploic and emissary veins to the brain and (2) precooling of arterial blood on-route to the brain by cooling venous blood in the facial and intranasal tissues and the surface of the head [26, 27]. In this study, we made use of the second mechanism by blowing cold air into the nasal cavities. Veins in the face, surface of the head, and nasal cavities drain into the cavernous sinus, a plexus of thin-walled veins, which closely intertwines with the internal carotid artery. This anatomical arrangement creates a favorable heat exchange mechanism for the transfer of heat from the warm blood in the carotid artery to the venous blood in the cavernous sinus.

Based on this heat exchange method, Dohi et al. achieved a lower cooling rate of ≈2.5 °C/h by blowing 24–26 °C air at a flow rate of 8–12 L/min directly into the nasal cavities of two patients with Foley catheters adjunct with surface cooling [28]. Recently, feasibility of preclinical intranasal evaporative cooling was successfully tested in patients after cardiac arrest [29, 30]. Castrén et al. demonstrated that the RhinoChill intranasal cooling device was effective to reduce tympanic temperature within the Pre-ROSC Intranasal Cooling Effectiveness trial (PRINCE trial) [31]. This method was able to show a significant decrease of tympanic temperature in the treatment group on arrival at hospital (34.2 versus 35.5 °C). The RhinoChill device vaporizes perfluorocarbon (PFC) along with oxygen at a flow rate of 60–80 L/min with a catheter system into the nasal cavity leading to a fast induction of hypothermia first to the brain as the main target organ and second to the body with a slight delay [31].

We showed that SBC can be achieved by blowing cold air at different flow rates into the nasal cavities. Both temperature and flow rate of the cold air generated by a vortex tube were controlled and monitored continuously by a controller that included a feedback system. By maintaining the air flow rate of 40–50 L/min at −3 ± 1 °C, the brain temperature dropped from 38.2 ± 0.7 °C to 34.0 ± 1.4 °C in 30 min while the core body temperature as measured by the rectal and esophageal temperature probes were >36 °C throughout the period. The maximum brain-esophageal temperature gradient of 3.9 ± 0.9 °C was reached about 40 min after the initiation of cooling and remained above 3 °C during the rest of intranasal cooling. Similarly, after the initial hour of rapid cooling, a brain-esophageal temperature difference of greater than 3 °C and esophageal temperature above 36 °C was maintained over 6 h by increasing the air temperature to 1 ± 3 °C and decreasing the flow rate to 30–50 L/min. Following 7 h of cooling, the brain was then allowed to gradually rewarm to the baseline temperature of 37.9 ± 0.4 °C in 2.5 h. All monitored physiologic variables except for HR, MAP, and CBF were unchanged from the baseline values throughout the cooling, maintenance, and rewarming phases. In our study, the magnitude of the difference between brain temperature (32–33 °C) and core body temperature (i.e., rectal and esophageal temperatures above 36 °C) kept above 3 °C to avoid complications of systemic hypothermia. However, the brain temperature can be cooled further using higher flow rate and lower air temperature.

The damage from cold air to the upper respiratory airway depends on the temperature, the level of ventilation, the duration of the exposure, and the susceptibility of the subject [32]. In this respect, it has to be noted that the quoted air temperature of −3 and 1 °C during the cooling and maintenance phases was measured at the output of the vortex tube. The temperature measured within the nasal cavity was 8 ± 2 °C higher than the temperature measured at the output of the vortex tube due to the known warming effect from the large surface area provided by the nasal conchae (turbinates) [33]. To investigate whether there was damage to the upper respiratory tract, MRI was performed before and after 7 h of cooling in three pigs. No nasal or nasopharynx mucosal swelling, necrosis, or hemorrhages were revealed on the MRI images, thus confirming the safety of blowing cold air into the nasal cavities. More detail histopathology examination may be warranted in future studies.

An important mechanism of hypothermia-induced neuroprotection is the preservation of brain adenosine triphosphate (ATP) levels from a reduction in cerebral metabolic rate of oxygen (CMRO_2_) [34]. As a consequence of the tight coupling between CMRO_2_ and CBF, many studies have shown that systemic hypothermia is associated with decrease in CBF [35, 36]. In this study, CBF fell below the baseline value as brain temperature decreased, remained suppressed throughout the rest of the cooling period, and only returned to the baseline level when the brain was rewarmed to the baseline temperature (Fig. 3f). In future experiments, we will investigate coupling between CMRO_2_ and CBF which is an important indicator of normal oxidative metabolism in the brain [37], by measuring both CBF using CT perfusion and CMRO_2_ using [15] O-PET scanning with a PET/CT scanner.

A reduction in HR and MAP was observed after the induction of intranasal cooling (Table 1). While the decrease in HR can be explained by the inotropic effect of mild hypothermia [38], the concomitant decrease in MAP is more likely due to the effect of propofol used for anesthesia [39]. Besides circulatory function, mild hypothermia may induce electrolyte abnormalities [40], more importantly cellular shift of potassium into the liver leading to hypokalemia [41].

In this study, there was a trend of increasing serum potassium level (i.e., hyperkalemia). The potassium level was maintained within the normal range during the experimental period by adjusting isoflurane concentration and propofol infusion rate together based on serum potassium level measurement at regular intervals. A possible explanation could be that only the brain was subjected to mild hypothermia (33.4 ± 0.3 °C) while the whole body temperature (36.6 ± 0.3 °C) was still within the normothermia range. With regard to administering potassium to correct hypokalemia, if observed, during hypothermia, potassium should only be administered to replace actual measured losses from gastrointestinal or urinary routes to prevent hyperkalemia and arrhythmias upon rewarming [42]. Furthermore, rewarming too quickly after hypothermia can cause dangerous electrolyte shifts, leading to potentially lethal arrhythmias [43]. Therefore, it is suggested that controlling the rewarming rate as low as 0.2–0.5 °C/h is preferred to reduce the neurological risks [13, 44].

This study has the following limitations. First, direct brain temperature measurements were made by a thermocouple thermometer implanted into the brain. Furthermore, invasive temperature probe can only provide a point measurement whereas it is important to know if temperature gradients exist in the brain with hypothermia induced by blowing cold air into the nasal cavities. With regard to this first limitation, MRI can measure brain temperature to within 1 °C [45]; but it is not a bedside method and cannot be used to monitor brain temperature at frequent intervals throughout the period of hypothermia that can last for a period of 12 to 72 h [32, 45]. Also, we had examined the temperature gradient within the brain of four pigs by measuring temperatures at the frontal and parietal lobe, and the gradient calculated as the difference was found to be no more than 0.1 °C. This could be due to the CSF circulation in the brain which minimizes temperature gradients if they exist. Furthermore, there are other several anatomical differences between pigs and human (e.g., cerebral blood flow, distance from the nasopharynx to the brain, ratio between the size of the nasal cavity to the brain) that are important in this context and may alter the results. With regard to the distance from the nasopharynx to the brain, we had examined the efficiency of intranasal cooling with a shortened nasal catheter (4–5 cm) for which the results were found to be similar to this study [46]. One important aspect which was not tested in our experiments is controlling the rewarming rate. Rewarming is a critical phase of therapeutic hypothermia in that too fast a rewarming rate may re-trigger destructive processes at the cellular level [47]. However, our method can also be used to control the rewarming rate and maintain normothermia in the post-rewarming period. In a recent study [46], we showed the potential use of our method to tightly control the rewarming rate within 0.3 ± 0.1 °C per hour by manually increasing the air temperature gradually and adjusting the flow rate on four juvenile pigs. In future studies, we will upgrade our controller to automatically adjust the flow rate and temperature of cold air according to the tympanic (ear drum) temperature measured by a near-infrared sensor in the ear canal as surrogate of the brain temperature. Moreover, in future studies, we will be blowing humidified air into the nasal cavities and humidity will be measured and controlled inside of the nasal catheter right before the nasal cavities. Histopathological studies on the lining of the nasal cavity will also be performed to demonstrate that there is no damage induced by blowing air for an extended period into the nostrils. As an initial step, we will obtain large animal data to prove that brain cooling with our fully automated device does provide brain protection in resuscitated cardiac arrest models in pigs.

## Conclusions

We have shown that blowing cooled air produced by a vortex tube into the nasal cavities is an effective and safe method to selectively reduce and maintain brain temperature on ten normal juvenile pigs. The cooling system was capable of achieving brain temperature of less than about 34 °C within 30–40 min and maintaining a brain-esophageal temperature difference of greater than about 3 °C over 6 h. Moreover, CBF values measured by CT perfusion decreased with decreasing temperature. Finally, all MRI images showed that there is no tissue damage following ~7 h of cooling using this technology.
